# Alternative Respiratory Pathway Component Genes (*AOX* and *ND*) in Rice and Barley and Their Response to Stress

**DOI:** 10.3390/ijms19030915

**Published:** 2018-03-20

**Authors:** Vajira R. Wanniarachchi, Lettee Dametto, Crystal Sweetman, Yuri Shavrukov, David A. Day, Colin L. D. Jenkins, Kathleen L. Soole

**Affiliations:** College of Science and Engineering, Flinders University of South Australia, GPO Box 5100, Adelaide, SA 5001, Australia; Vajira.Wanniarachchi@anu.edu.au (V.R.W.); dame0006@flinders.edu.au (L.D.); Crystal.Sweetman@flinders.edu.au (C.S.); Yuri.Shavrukov@flinders.edu.au (Y.S.); David.Day@flinders.edu.au (D.A.D.); Colin.Jenkins@flinders.edu.au (C.L.D.J.)

**Keywords:** alternative oxidase, NADH dehydrogenase, rice, barley, oxidative stress

## Abstract

Plants have a non-energy conserving bypass of the classical mitochondrial cytochrome c pathway, known as the alternative respiratory pathway (AP). This involves type II NAD(P)H dehydrogenases (NDs) on both sides of the mitochondrial inner membrane, ubiquinone, and the alternative oxidase (AOX). The AP components have been widely characterised from Arabidopsis, but little is known for monocot species. We have identified all the genes encoding components of the AP in rice and barley and found the key genes which respond to oxidative stress conditions. In both species, AOX is encoded by four genes; in rice *OsAOX1a*, *1c*, *1d* and *1e* representing four clades, and in barley, *HvAOX1a*, *1c*, *1d1* and *1d2*, but no *1e*. All three subfamilies of plant *ND* genes, *NDA*, *NDB* and *NDC* are present in both rice and barley, but there are fewer *NDB* genes compared to Arabidopsis. Cyanide treatment of both species, along with salt treatment of rice and drought treatment of barley led to enhanced expression of various AP components; there was a high level of co-expression of *AOX1a* and *AOX1d*, along with *NDB3* during the stress treatments, reminiscent of the co-expression that has been well characterised in Arabidopsis for *AtAOX1a* and *AtNDB2*.

## 1. Introduction

The alternative respiratory pathway of plant mitochondria is an important bypass of the energy-conserving classical electron transport (ETC) pathway [1]. In the classical ETC, oxidation of mitochondrial NADH occurs via rotenone-sensitive Complex I and cytochrome *c* (cyt *c*) oxidase, concomitantly generating a proton motive force that drives ATP synthesis via ATP synthase. Plants have a non-phosphorylating (non-energy conserving) bypass of the cyt *c* pathway, evading adenylate control of cellular redox poise [2,3,4]. This bypass, known as the alternative respiratory pathway (AP), involves rotenone-insensitive type II NAD(P)H dehydrogenases (NDs) on both the outer and inner surface of the mitochondrial inner membrane, ubiquinone, and the alternative oxidase (AOX). Activity of the AP, however, does not generate a proton gradient and excess energy is released as heat. There is strong evidence that operation of the AP prevents over-reduction of the ETC [5,6,7,8], thereby minimizing the generation of reactive oxygen species (ROS) and allowing a degree of metabolic flexibility in the cell. A large number of publications have reported that this pathway responds to temperature, nutrients, heavy metals, high light, drought and oxidative stress [4].

In Arabidopsis, members of the AP include seven type II NDs (NDB1–4, NDA1–2, and NDC1) and five AOXs (AOX1a–d and AOX2) [9]. NDB1-4 have been localized to the external face of the mitochondrial inner membrane, while NDA1 and 2, and NDC1 were determined to be internal (facing the matrix), based on import assays [10,11]. Transcript, protein and activity levels of NDs and AOX appear to be regulated in a tissue, developmental, and stress-inducible manner [11,12,13,14,15]. This suggests specialization of function and possible formation of specific ND/AOX pathways in response to particular stimuli and there is evidence that an alternative electron transport chain of NDA2, NDB2 and AOX1a forms in Arabidopsis during many stresses [14].

The majority of studies to date on the response of the AP to stresses have focussed on Arabidopsis and other dicot plants, while there have been few studies on monocots, including crops such as rice and barley. In rice, *Oryza sativa*, Ito, et al. [16] first identified *OsAOX1a* and *OsAOX1b* isoforms, which were up-regulated in whole seedlings during cold stress. Considine, et al. [17] and Saika, et al. [18] identified a third isoform *OsAOX1c*, which showed developmentally regulated expression patterns and was not stress-responsive. Considine, et al. [17] also identified an additional gene *OsAOX1d* that was thought to be not expressed, but more recently Costa, et al. [19] reported a number of corresponding ESTs found in germinating tissue. *OsAOX* genes have been shown to respond to different stresses such as cold, drought, dehydration, and salt [16,20,21,22,23], but not all studies have assessed all three isoforms and very few have assessed the corresponding protein and activity levels. In contrast, while rice orthologues for the ND family have been described [3,24] there have been no studies on this gene family’s response to stress.

Even less is known about the AP components in barley, *Hordeum vulgare*. The *HvAOX* gene family has been described by Costa et al. [19], identifying four isoforms, *HvAOX1a*, *HvAOX1c*, *HvAOX1d1* and *HvAOX1d2*, but the orthologues/homologues of the *ND* family are as yet unidentified. There are two reports showing that *AOX* responds to stress in barley. An *AOX*-like gene in *H. spontaneum* (wild barley) was up-regulated in leaves during cold stress, and *HvAOX1a* in *H. vulgare,* cv. Golden Promise during short-term heat stress in spikes at anthesis, and AOX protein levels were up-regulated under nutrient stress and UVB stress in highland barley [25,26]. Clearly, the stress-responsiveness of AP components needs further examination in this important crop.

It has been suggested that *AOX* sequence variability has the potential to be used as a marker for selective breeding [27], but it is likely that other components such as the *ND*s, which are required for better oxidative stress responses, also need to be considered. *AOX* has been well studied in many species, especially in Arabidopsis, but the *ND*s have received less attention. In this study, we expand these studies into the cereal crops, rice and barley, identifying the alternative respiratory pathway components in *O. sativa* and *H. vulgare*, and investigate the expression of *AOX* and *ND* genes in different tissue types at both the transcript and protein level under mitochondrial ETC-inhibiting and ROS-generating conditions. The results provide a platform for investigations of these genes as potential selective markers for rice and barley breeding.

## 2. Results

### 2.1. Identification of the Genes Encoding Alternative Respiratory Pathway (AP) Components in Rice and Barley

#### 2.1.1. Alternative Oxidase

Previous studies have identified the gene family encoding *AOX* in rice [16,17,18] and recently Costa et al. [19] have reclassified these genes as *OsAOX1a*, *OsAOX1c*, *OsAOX1d* (previously *OsAOX1b*) and *OsAOX1e*. This nomenclature was based on the fact that *OsAOX1b* was more like an orthologue of the eudicot *AOX1d* gene. Sequence searching (BLAST) of the Rice Genome Annotation Project database (http://rice.plantbiology.msu.edu) identified four gene sequences homologous to Arabidopsis: *OsAOX1a* (LOC_Os04g51150), *OsAOX1d* (LOC_Os04g51160), *OsAOX1c*, (LOC_Os02g47200), and *OsAOX1e* (LOC_Os02g21300) in agreement with Costa et al. [19]. Homologous sequences for all *OsAOX* genes identified in japonica rice were also present in indica rice (Table S1). Pairwise alignment of CDS sequences revealed that all *OsAOX* isoforms from japonica rice were very similar or identical to those of indica rice (Table S1) consistent with them being closely related.

**Figure 1:**
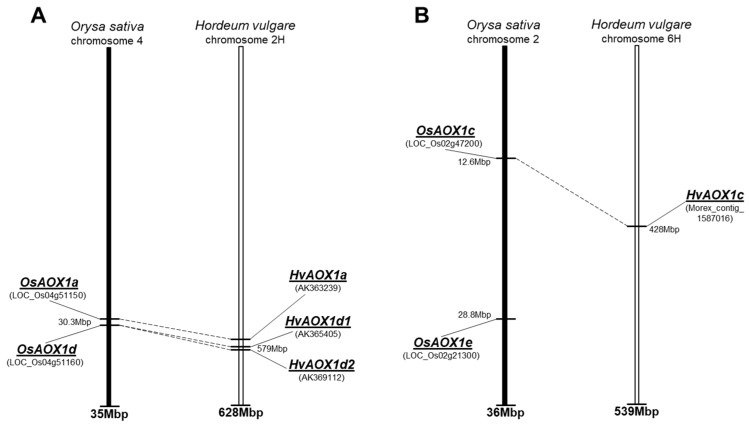
Rice and barley comparative maps with positions of identified *AOX* genes. (**A**) *AOX1a* and *AOX1d*; (**B**) *AOX1c* and *AOX1e*. Corresponding genes are indicated by dashed lines. Information about rice and barley genes are their locations was extracted from web-sites, respectively: http://rice.plantbiology.msu.edu and http://pgsb.helmholtz-muenchen.de/plant/barley.

The isoforms of *HvAOX* in barley, *H. vulgare*, were recently reported in the *AOX* phylogeny study of Costa et al. [19], where four *AOX* partial genomic sequences were identified from a few ESTs. Prior to this, only one report of an *AtAOX1a*-like gene had been identified during a microarray analysis of high temperature stress in barley floral tissue [28]. To confirm these findings, the three cDNA sequences of *OsAOX* (Table S1) were used as queries to conduct BLASTN searches within *H. vulgare* mRNA sequence databases, using NCBI (https://www.ncbi.nlm.nih.gov) and IPK (http://webblast.ipk-gatersleben.de/barley_ibsc); four putative cDNAs were found in *H. vulgare*, cv. Haruna Nijo (Table 1). These were of similar size except for *HvAOX1c*. The putative *HvAOX* cDNA sequences were used to conduct BLASTN search against the assembly WGS-Morex database on IPK to confirm correct identification. Genomic contig sequences from cv. Morex demonstrate high sequence identity with sequences identified by Costa et al. [19] and provide an estimation of chromosomal location (Table 1). Two of the rice genes are located on the same chromosome, where a common tandem position for *OsAOX1a* and *OsAOX1d* genes was identified in rice chromosome 4 (Figure 1A, [19]). A strong colinearity with rice chromosome 4 was found in barley chromosome 2H with an additional likely duplication leading to tandem repeat *HvAOX1d1* and *HvAOX1d2* in close proximity on the chromosome (Figure 1A). In contrast, two other *AOX* genes in rice, *OsAOX1c* and *OsAOX1e*, were localised on different arms of rice chromosome 2 (Table S1), while only a single *HvAOX1c* was identified on barley chromosome 6H, in a strongly colinear genetic region to those for rice *OsAOX1c* (Figure 1B, Table 1). No barley gene was identified homologous to rice *OsAOX1e*.

#### 2.1.2. Type-II NADH Dehydrogenase Family

**Figure 2:**
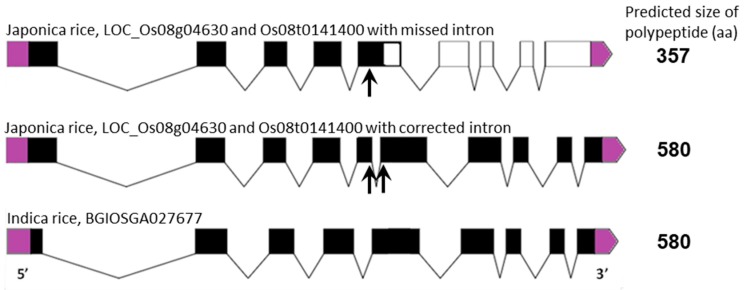
Identification of an additional intron in the sequence of *OsNDB3*, japonica rice, which encodes a predicted OsNDB3 polypeptide of 580 aa, correcting the annotated size of 357 aa. A schematic presentation of the *OsNDB3* gene structures in both japonica and indica rice with the missing or corrected intron. Exons are shown as black boxes or as clear boxes after the premature stop-codon of the missed intron. Start-codons on 5′-end and stop-codons on 3′-end are shown in purple. The predicted sizes of the polypeptides are indicated.

The gene family of Type-II NAD(P)H dehydrogenases in plants is divided into three subfamilies: *NDA*, *NDB* and *NDC* [10,29], with NDA and NDC proteins located on the inside of the inner mitochondrial membrane, facing the matrix space, and NDB proteins on the outside of the inner membrane, facing the intermembrane space [11]. Orthologues of these genes in rice have been identified previously [3,24] and their designations correspond to the gene sequences used in this study (Table S1). Sequence alignment studies showed that, similarly to the *AOX* genes, the *ND* genes from japonica rice are nearly identical to those from indica rice except for NDB3, which in japonica appears to have a shorter sequence compared to that in indica rice. An analysis of the gene structure for rice *ND* genes shows that generally they contain a similar number of exons, however the intronic regions are often longer than members of the *ND* gene family in Arabidopsis [29]. Importantly, the discrepancy between japonica and indica can be explained by an additional intron present in exon 5 in the japonica *OsNDB3* gene, which has been documented incorrectly in current databases (http://rice.plantbiology.msu.edu/cgi-bin/ORF_infopage.cgi) as a coding region. The reading of this intronic region as coding sequence results in a premature termination codon, giving a 357 aa protein rather than the 580 aa protein predicted from the indica *OsNDB3* gene (Figure 2 and Figure S1). Evidence for this insertion region being an intron comes from its absence in rice ESTs found in NCBI for this region of the genome.

The *O. sativa ND* cDNA sequences (Table S1) were used to conduct BLASTN searches on NCBI revealing 6 barley *ND* sequences (Table 1). A BLASTP search of the predicted *O. sativa* protein sequences against NCBI and IPK databases revealed the corresponding protein sequences. All *ND* genes are predicted to encode proteins in the range of 54 to 65 kDa, similarly to Arabidopsis. Chromosome location was also identified for these genes (Table 1).

**Figure 3:**
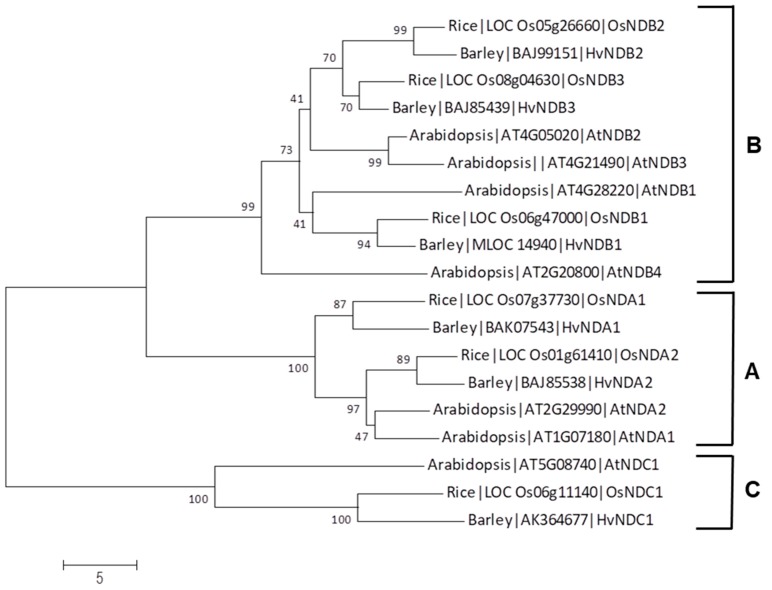
Relationship tree that shows the sequence homology of alternative dehydrogenases from rice, barley and Arabidopsis. Clades A, B and C correspond to NDA, NDB and NDC, respectively. The tree was constructed using the Neighbour-Joining method in MEGA 7. Numbers at each node are the percentage bootstrap values of 1000 replicates. The scale bar indicates the number of amino acid substitutions at each site.

An analysis of the sequence identity between Arabidopsis and rice *ND* genes revealed that *OsNDB2* and *OsNDB3* have highest identity with *AtNDB3* (Table S2); however, a previous study [24] used *OsNDB2* notation for the gene (LOC_05g26660), so we have retained this designation here. A relationship tree of sequences from Arabidopsis, rice and barley grouped the corresponding proteins into three clades, NDA, NDB and NDC, but it also confirmed the close sequence relationship between NDB2 and NDB3 in both rice and barley (Figure 3). No orthologue for AtNDB4 was apparent in these monocots.

### 2.2. Subcellular Localisation and Structural Similarities

Using a bioinformatics approach, the subcellular location of rice and barley AOX was confirmed to be mitochondrial. The situation with the ND proteins is not so clear. Arabidopsis NDs contain signals in their sequences that can target these proteins to subcellular compartments other than the mitochondria; for example, AtNDC1 can be targeted to both the chloroplast and the mitochondrion [30]. More recently, Xu et al. [24] indicated that OsNDB1 and OsNDB2 contain both mitochondrial and peroxisomal (PTS1) targeting signals, and showed that when the C-terminal sequence of these proteins was fused to GFP they were targeted to the peroxisome in vivo. OsNDB3 was not included in that study and so its location remains uncertain. We used a number of bioinformatic targeting programs to reassess the putative subcellular location of all the rice and barley ND proteins (Table S3); all contained mitochondrial targeting information and HvNDB1 and HvNDB2, like their rice counterparts, were predicted also to contain a PTS1 sequence, a small amino acid sequence found at the C-terminal end of peroxisomal proteins [11,30]. Since the rice proteins have been demonstrated to be targeted to the peroxisome [24], it is likely that this is also true for HvNDB1 and HvNDB2. Interestingly, OsNDB3 and HvNDB3 were not predicted to target to the peroxisome, reminiscent of AtNDB2 and AtNDB4 that are also confined to the mitochondria [30].

AOX proteins are characterised by conserved cysteine residues (Cys_I_ and Cys_II_), four helical regions, and conserved glutamine and histidine residues that act as iron ligands [31]. The cysteines have been linked to post-translational regulation of AOX, being involved in the formation of an inactive dimer via a disulphide bond, which when reduced allows the enzyme to be active ([31] and references therein). Cys_I_ and Cys_II_ [31,32,33,34] and more recently Cys_III_ [35], have been shown also to be important in the response of these AOX isoforms to metabolites that activate AOX, such as pyruvate and glyoxylate [36]. As previously reported, OsAOX1d (formerly OsAOX1b) has a serine substitution at Cys_I_ and Cys_II_ positions [16], indicating that it cannot form a covalent disulphide-linked dimer and therefore may not be inactivated in vivo. However, it may have a rather low activity. When AtAOX1a was mutated to replace Cys_I_ with a serine, it no longer responded to pyruvate but was instead activated by succinate [34,35]. This is also true of a naturally occurring tomato AOX [37]. However, when serine was substituted for Cys_I_ in AtAOX1c or d, succinate did not stimulate [35], presumably because of other sequence differences in these proteins. Whether OsAOX1d is activated by succinate is not known and has to be experimentally determined before we can judge whether it is regulated in vivo.

**Figure 4:**
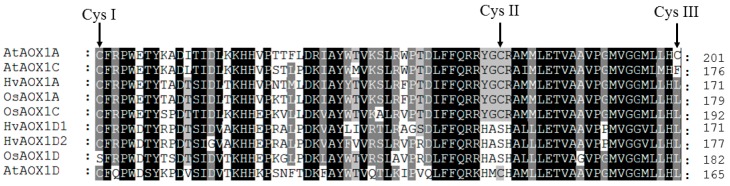
Alignment of a section of AOX isoforms in rice and barley. The amino acid sequences were aligned using the Clustal program. The region showing all the cysteine residues of these AOX proteins is shown. OsAOX1d has a serine at Cys_1_; HvAOX1d1, HvAOX1d2 and OsAOX1d have a serine at Cys_II_; all rice and barley AOX isoforms have a leucine at Cys_II_ instead of a cysteine or phenylalanine.

Interestingly, while no AOX isoform in barley has a serine substitution at the Cys_I_ position, HvAOX1d1 and HvAOX1d2 have serine at the Cys_II_ position. This is not expected to affect the enzyme’s regulation by dimerization via oxidation-reduction, but it may affect its stimulation by glyoxylate [31]. All AOX isoforms in rice and barley have leucine at the Cys_III_ position, which is similar to AtAOX1d, not AtAOX1a (Figure 4).

HvAOX1d1 and 2 are particularly interesting because they have a C, S, L (Cys_I_, Cys_II_, Cys_III_) configuration (Figure 4). This configuration has not been reported for any other AOX protein to date, but when AtAOX1d was mutated to a C, S, L form, its basal activity (i.e., without an organic acid activator) increased quite dramatically [35]. It is possible, therefore, that the HvAOX1d isoforms are also super active, but again, this needs to be experimentally proven in a heterologous assay system.

**Figure 5:**
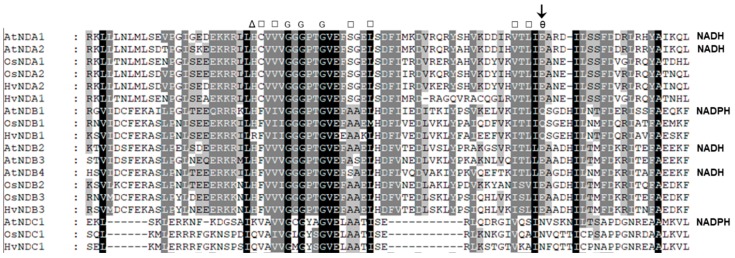
An alignment of the NAD(P)H binding domain in rice and barley ND families with Arabidopsis homologues. The sequences were aligned using the Clustal program. Substrate specificity is given on the right for each enzyme that has been experimentally determined [10,39,42]. Residues are Δ, basic or hydrophilic; □, hydrophobic; ө, acidic; and G, Glycine. The acidic residue alters the specificity for NADH- and NADPH-specific proteins and is indicated as an arrow.

An alignment of the rice and barley ND protein sequences showed that they all contain both of the conserved dinucleotide folds (DNF) that are involved in Flavin and NAD(P)H binding. It has been shown that the DNF sequence at the C-terminal end of these proteins is involved in NADH and NADPH binding and an analysis of this DNF structure, which has a βαβ motif, has shown that a residue at the end of the second β-pleated sheet can influence substrate specificity and the key residues involved have been denoted in Figure 5 [38]. A glutamate (E) in this position enables NADH binding and repels the phosphate on NADPH. Isoforms that show specificity for NADPH have either an uncharged glutamine (Q) or asparagine (N) at this position. These residues have been experimentally confirmed to be involved in NADPH binding via recombinant expression of the Arabidopsis NDs [39]. On this basis, a structural analysis of the rice and barley ND sequences suggests that NDB1 and NDC1 are NADPH specific, while all the others are likely to be NADH specific (Figure 5). Recently, it has been shown that *AtNDC1* encodes an enzyme involved in vitamin K synthesis in the chloroplast [40]. Its role in mitochondria is unknown, but it is targeted to the matrix side of the IM and may be the protein responsible for matrix NADPH oxidation, detected in some plant mitochondria [41].

The NDB proteins, when aligned with NDAs and NDC, contain additional amino acid sequence in which are located EF-hand motifs that have been linked to calcium binding. The conserved N-terminal EF-hand motif has been shown to be conserved in Arabidopsis NDB where it has been experimentally demonstrated to bind calcium [39,43]. All rice and barley NDBs contained this EF hand, which suggests that these dehydrogenases may be regulated by calcium. In Arabidopsis, AtNDB4 lacks critical key residues in its EF-hand motif and has been shown to not bind calcium [39], but rice and barley do not appear to have this calcium-independent NDB protein (Table 1, Figure 2).

### 2.3. Expression of AP Components under Chemical-Induced Stress

**Figure 6:**
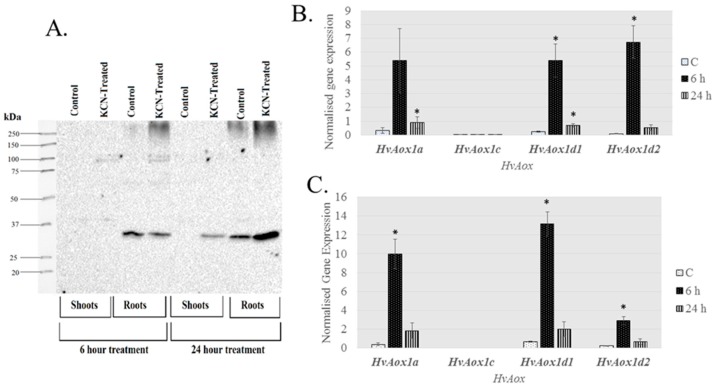
Chemical treatment increases AOX protein levels in barley shoot and root tissue. Barley seedlings (14 days old) were exposed to 5 mM potassium cyanide (KCN) for up to 24 h, then root and shoot tissue were used for crude protein extractions. (**A**) Immunoblot of crude protein extracts under reduction conditions. The expected size for HvAOX is 35–37 kDa. Expression levels of *AOX* isoforms were determined using qRT-PCR in shoots (**B**) and roots (**C**). Expression data were normalized with reference genes *Actin*, *Ubiquitin* and *Pdf* (Protein phosphatase) and shown as mean of 4 biological replicates. Gene expression data were derived from the same experiment as presented in Table 3, but shown as normalised expression instead of fold-change, to demonstrate the relative level of expression between genes. * denotes significantly different (*p* < 0.01) from the control (**C**).

A focus of this work was to determine the stress responsive components of the AP in the monocots rice and barley. Initially, plants were exposed to KCN and transcript abundance of AP component genes determined and compared to untreated controls. KCN inhibits cytochrome oxidase leading to an over-reduction of the electron transport chain and accumulation of ROS [44]. In this study, *OsAOX1e* expression was not assessed. While Costa et al. [19] found a small number of ESTs with partial sequence corresponding to this gene, they were isolated from germinating seeds and are more likely to be linked to development. In untreated tissues, *OsAOX1c* was the most highly expressed *AOX* gene in shoots but not roots (Table S4). In contrast, in untreated barley, *HvAOX1c*, was the lowest expressed gene in both shoots (not detectable) and root tissue (Table S4). After 6 h of KCN treatment, all *AOX* isoforms in both rice and barley increased in expression in both shoots and roots, except for *AOX1c* (Table 2 and Table 3). In KCN-treated rice, *OsAOX1d* was the most responsive of the AP genes, in both roots and shoots. *OsAOX1a* transcript returned to lower levels 24 h after treatment, whereas the increase in expression was sustained for *OsAOX1d* in shoots (Table 2). *HvAOX1a*, *HvAOX1d1* and *HvAOX1d2* were expressed at similar levels in control roots and shoots (Table S4), and were very responsive to KCN treatment, but this was not sustained at 24 h in any tissue (Table 3 and Figure 6). An analysis of AOX protein levels in barley tissue total protein extracts showed that AOX protein was detectable in control root samples, but not in shoot tissue. However, AOX protein increased in both shoot and root tissue after 24 h exposure to KCN (Figure 6).

Prior to KCN treatment, *OsNDA1*, *OsNDB*2, *OsNDB3* were the most highly expressed of the *ND* genes in both roots and shoots (Table S4). In contrast, in untreated barley tissue the most highly expressed *ND* genes were *HvNDA2*, *HvNDB3* and *HvNDB1*, while *HvNDA1* was not detectable (Table S4). In rice, *OsNDA1* and *OsNDB3* expression was upregulated within 6 h of KCN treatment in shoots and roots, but was not sustained at 24 h. *OsNDB2* was upregulated in shoots after 6 h and in root tissue after 24 h. *OsNDA2* expression also increased in roots (Table 2). After KCN treatment, *HvNDA2* and *HvNDB3* were upregulated in shoots and roots and *HvNDB2* to a lesser extent in shoots after 6 h (Table 3). Again, the *HvNDB2* response was sustained in shoots at 24 h. *HvNDA1* was not detectable in control tissue, but showed a little expression, at the limit of detection after KCN treatment, therefore a fold-change could not be calculated although this gene may also be stress-responsive (Table 3).

### 2.4. Expression of Rice and Barley AP Components under Abiotic Stress

**Figure 7:**
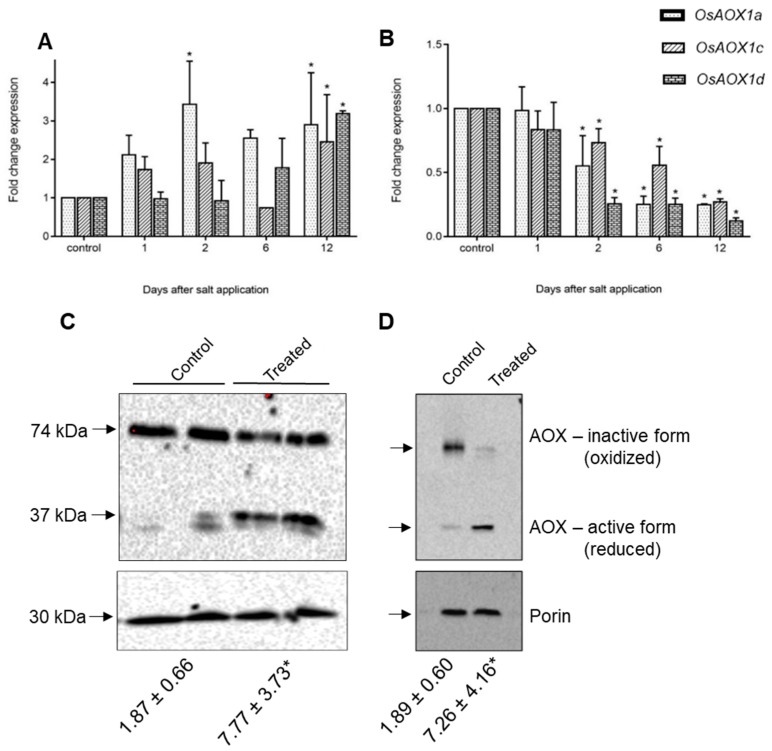
Response of alternative oxidases (AOXs) in rice under salt stress. Fold change expression of *AOX* isoforms, *AOX1a*, *AOX1c* and *AOX1d* in seedling shoots (**A**) and roots (**B**) were analysed in response to 120 mM NaCl using qRT-PCR over a period of 12 days. Data are shown as the mean ± SE of three biological replicates relative to the control at each time point set as 1.0. Significant differences are indicated by asterisks (*) at *p* < 0.01 (*t*-test). Immunoblots of the AOX protein present in purified mitochondria from salt-treated and non-treated (control) shoots (**C**) and roots (**D**) harvested 9 days after the start of salt application. The numbers below each lane of the blot, represent the AOX capacity determined for the purified mitochondria for that treatment.

Data from Genevestigator show that the same stress-responsive genes identified in the KCN treatment in rice are up-regulated under many abiotic and biotic stresses, including cold, drought, nutrient stress, and arsenate treatment (Table S5). Interestingly, in the salt studies available, *OsAOX1a*, *OsAOX1d*, *OsNDB3* and *OsNDB2* showed the highest up-regulation in shoot tissue, but were down-regulated in root tissue. We explored this further in rice exposed to 120 mM NaCl over a 12 days period to examine the specific AP response. In shoots, *OsAOX1a*, was up-regulated early in the stress while *OsAOX1d*, showed a later response and *OsAOX1c* was only up-regulated at 12 days (Figure 7A). In the roots, there was a decline in the abundance of all AOX isoform transcripts after the stress (Figure 7B). To elaborate this response further, mitochondria were isolated from shoot and root tissue at 9 days after salt application and respiratory activities determined. In both shoot and root mitochondria there was an increase in AOX capacity. In shoots this was reflected in an increase in AOX protein, especially as the monomeric active form (Figure 7C). The two bands present may reflect different gene products, but only N-terminal sequencing would confirm this. However, in soybean, different apparent Mr of AOX proteins in immunoblots was not indicative of different size proteins [45] In root mitochondria, overall AOX protein abundance did not change but there was a shift from the oxidised dimer to the monomeric form, consistent with the increase in AOX capacity. All the data for roots are consistent with the view that in response to salt stress, existing AOX protein is activated post-translationally, while in shoots there may be an increase in overall AOX protein as well as a post-translational activation.

**Figure 8:**
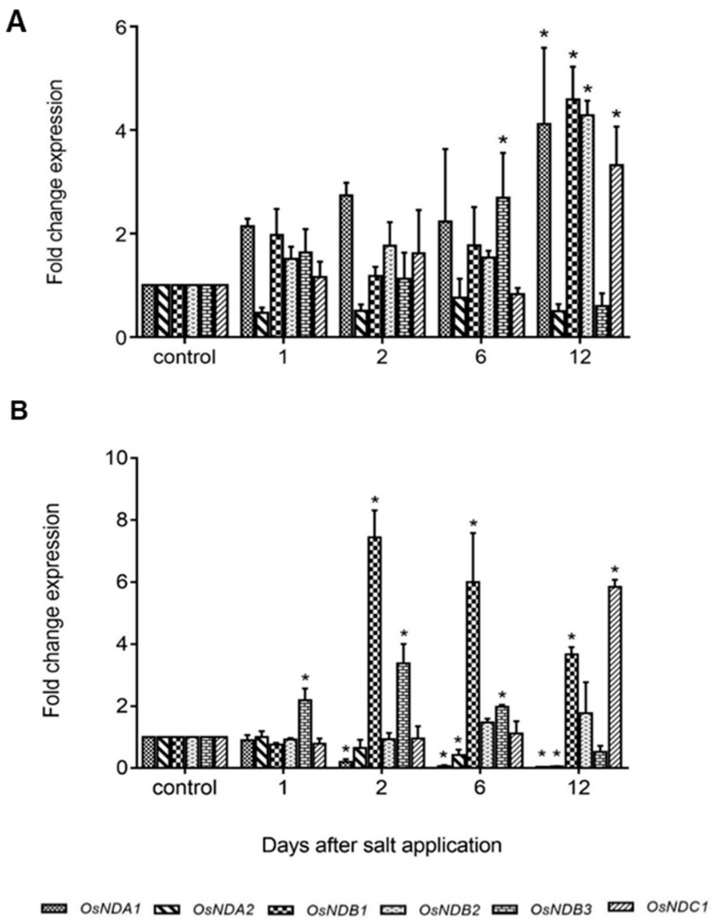
Expression of alternative dehydrogenases (NDs) in rice under salt stress. Fold change expression of *ND* isoforms, *OsNDA1*, *OsNDA2*, *OsNDB1*, *OsNDB2*, *OsNDB3* and *OsNDC1*, in seedling shoots (**A**) and roots (**B**) were analysed in response to 120 mM NaCl using qRT-PCR over a period of 12 days. Data are shown as the mean ± SE of three biological replicates relative to the control at each time point set as 1.00. Significant differences are indicated by asterisks (*) at *p* < 0.01 (*t*-test).

**Figure 9:**
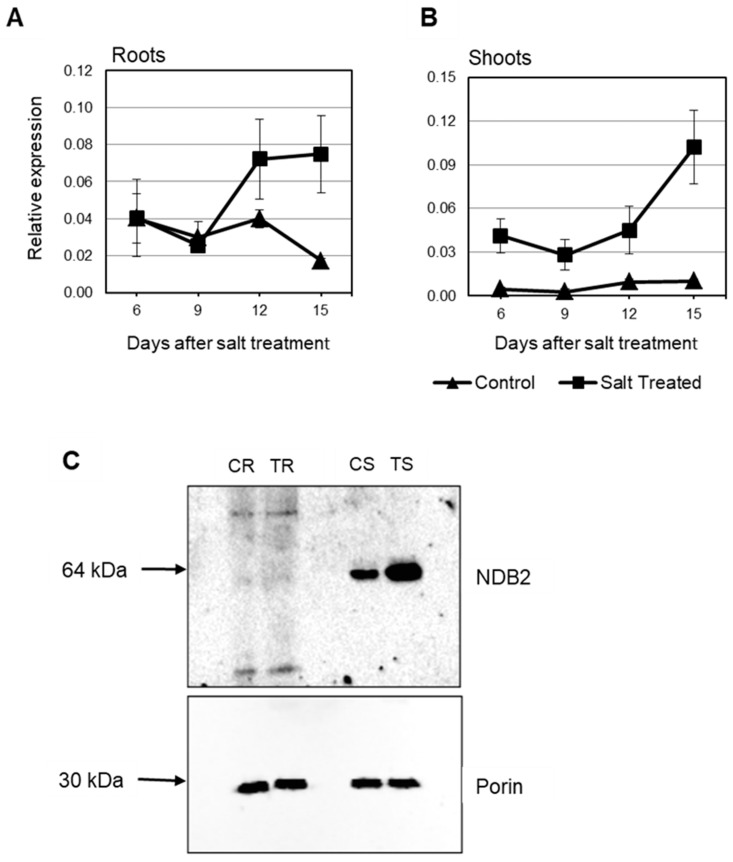
Expression of *OsNDB2* in rice under salt stress. Expression was analysed in seedling roots (**A**) and shoots (**B**) exposed to 120 mM NaCl over the period of 15 days using qRT-PCR. Data are shown as the mean relative gene expression ± SE of three biological replicates; (**C**). An immunoblot of the OsNDB2 protein present in mitochondria isolated from salt-treated and control shoots harvested 9 days after the start of salt application. CR-control roots; TR-treated roots; CS-control shoots; TS-treated shoots.

In this study, in shoots from salt stressed plants, *OsNDB3* was the first *ND* gene to be up-regulated (day 6) while by day 12, *OsNDA1*, *OsNDB1*, *OsNDB2* and *OsNDC1* were all up-regulated, in agreement with Genevestigator data (Figure 8; Table S5). In roots, *OsNDB3* was up-regulated at the earliest time-point, followed by *OsNDB1* at day *2* and *OsNDB2* at day 6 and *OsNDC1* at day 12 (Figure 8). Further, there was a significant decrease in *OsNDA1* and *OsNDA2* levels in roots, reminiscent of the *OsAOX* gene response in root tissue. It is unknown whether this change in *OsNDA* transcript levels reflects a change in corresponding protein levels or activities. Antibodies for each ND in rice or barley are currently not available, but an antibody raised to a peptide-specific for the *OsNDB2* isoform was developed and used to detect NDB2 protein in mitochondria isolated from shoot and root tissue. OsNDB2 protein was detectable in control shoot but not in root mitochondria (Figure 9C). There was an increase in OsNDB2 protein in mitochondria from day 9 salt treated shoot tissue but not root mitochondria, consistent with transcript data collected for this tissue (Figure 9A,B).

**Figure 10:**
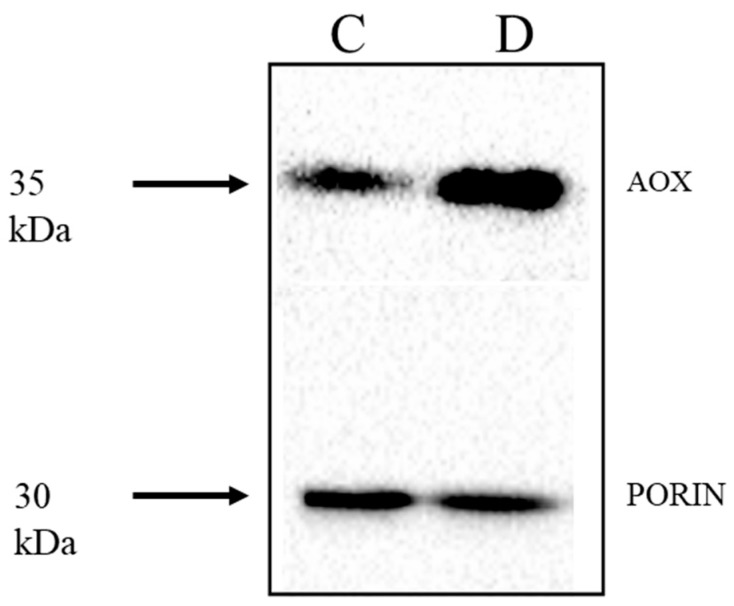
Detection of AOX protein in barley exposed to moderate drought stress. Soil grown 12 days old barley seedlings (*Hordeum vulgare*, cv. Golden Promise) were exposed to drought stress by withholding water for 14 days under greenhouse conditions. AOX protein was detected in total protein extracts of shoot tissue in plants grown in well-watered controls (C) and drought, withhold conditions (D) resolved under reducing conditions. The relative water contents for the plant were 83% and 69% in control and drought conditions, respectively.

Genevestigator data showed that the same stress-responsive genes identified in the KCN treatment of barley were also up-regulated by many abiotic and biotic stresses (Table 3 and Table S6). *HvAOX1a, HvAOX1d1* and *HvAOX1d2* were the stress-responsive *AOX* isoforms, along with *HvNDB3*. No information for *HvNDB2* is available in Genevestigator. Interestingly, *HvNDA1* was reported to increase in some perturbations, but as the *HvNDA1* and *HvNDA2* show high sequence identity, it is possible that there may be a mis-identification of these genes in Genevestigator. Increases in abundance of AP gene transcripts occur with biotic stress in barley and are also seen in abiotic stresses such as cold and drought. To elaborate on the response to drought, total protein extracts were made from shoot tissue of barley grown under normal or moderate drought conditions and probed with the AOX antibody. Shoots from drought treated plants had higher AOX protein levels than control samples, indicating transcriptional and/or translational control (Figure 10). Since these protein extracts were obtained under reducing conditions, it is not clear if there is also a post-translational level of activation via disulphide reduction under these conditions, and this is worth further investigation.

It is well documented that *AtAOX1a* and *AtNDB2* show a high level of co-expression under various stress conditions [14]. Transcriptomic datasets for perturbations in Genevestigator were scanned to determine the genes showing the highest correlation of expression with *AtAOX1a* and *AtNDB2* as well as all rice and barley AP genes. Table S7 shows the level of correlation between AP genes. For example, *AtNDB2* is the sixth most correlated gene expressed with *AtAOX1a*, confirming the close co-expression relationship between these genes during stress. Interestingly, *AtNDA2* is also highly expressed with *AtNDB2* and *AtAOX1d* lies within the top 100 genes for co-expression with *AtAOX1a*.

A similar analysis of rice and barley AP genes suggests that co-expression of *AOX* and *ND* genes also occurs in monocots. In both rice and barley, the genes with strongest correlated expression are *AOX1a* and *AOX1d* (*HvAOX1d1* and *HvAOX1d2*, in barley). In rice, *OsNDB3* and *OsNDA1* are the isoforms most highly co-expressed with *AOX* isoforms. For barley, a close co-expression relationship exists between *HvNDB3, HvAOX1d1* and *HvAOX1d2* (Table S7).

## 3. General Discussion

Plant mitochondria contain a complex electron transport chain, characterised by ND and AOX proteins that constitute an alternative pathway, AP, which is not linked to energy conservation. Evidence suggests that the AP plays a role in plant abiotic stress tolerance through minimising ROS [44,46], influencing stress related amino acid synthesis [47], protecting cytochrome oxidase from ROS under photoinhibitory conditions, such as drought [48], and influencing stomatal function [49].

Many studies that have examined the role of the AP in plant growth and stress responses have focused on AOX, with the NDs often overlooked, and have rarely used monocot species. In this study, we have identified all the genes associated with the components of the AP in rice and barley, examined their expression under stress and identified the key genes involved in stress response.

As described previously, AOX is encoded by four genes in rice and barley, all clustering within the *AOX1-like* gene clade and none in the *AOX2-like* gene clade [19]. Recently, *AOX2-like* genes have been found in ancient monocot ancestors [50], but they are yet to be found in more recent monocots, such as rice. *OsAOX1a* and *OsAOX1d* were found to be located in a tandem arrangement on the same chromosome, and we now show that this section of the rice genome maps to a similar region in barley where a gene duplication has resulted in formation of *HvAOX1d1* and *HvAOX1d2*, which are closely related in sequence. These closely located genes are highly co-expressed in both rice (*OsAOX1a* and *OsAOX1d)* and barley (*HvAOX1a*, *HvAOX1d1* and *HvAOX1d2*) in the stress experiments in this study and in previous studies reported in Genevestigator data. While this co-expression was evident in previous studies where rice plants were exposed to stresses such as chilling, drought and salinity [20,22,51], this is the first report of this response for barley (Tables S6 and S7). *AOX1c* was not stress responsive in either rice or barley in our experiments. In other species there are some *AOX1* genes that do not respond to stress (e.g., *AtAOX1b* and *1c* in Arabidopsis, based on observations from the Genevestigator Perturbations data set), so this is not unique. However, the level of expression of *AOX1c* differed greatly between the two species. *OsAOX1c* was constitutively expressed in both shoot and root tissue of rice. In contrast, *HvAOX1c* was expressed at very low levels in shoots and was not detectable in roots of 12 days old barley plants, suggesting a species difference in the regulatory elements present in the promoter regions of this gene and tissue-specific roles not yet uncovered.

We also report for the first time a preliminary characterisation of the barley *ND* genes. All three subfamilies of plant *ND* genes, *NDA*, *NDB* and *NDC* are present in both rice and barley, but both species have fewer *NDB* genes than the well characterised Arabidopsis. The amino acid sequence can be a guide to substrate specificity of ND proteins. On this basis, apart from NDB1 and NDC1, all the rice and barley NDs apparently use NADH as a substrate, as described for Arabidopsis [39]. All the NDBs we have identified contain a conserved EF hand motif and so have the potential to be regulated by calcium binding, but rice and barley lack an equivalent to AtNDB4, an NADH-specific ND that does not bind calcium. The role of this protein in Arabidopsis has not yet been clarified, but its absence in rice and barley suggests a metabolic difference between these species. Further analysis revealed that the barley and rice NDB3 proteins lack the peroxisomal targeting sequences previously identified for OsNDB1 and OsNDB2 [24], suggesting that NDB3 in rice and barley is orthologous to AtNDB2.

Experiments with chemical and abiotic stress treatments show that a number of *ND* genes in both rice and barley are responsive to oxidative stress along with AOXs. The most stress-responsive of these genes was *NDB3* in both monocots, but *NDA1* or *2* and *NDB2* also responded. However, *NDB2* showed a different temporal response during KCN and salinity treatment of rice with lower, but sustained expression, possibly reflecting a response to different signals during the stress. Experiments with an antibody specific for OsNDB2 showed that the protein also increased in roots of stressed plants. Data in Genevestigator confirms the transcriptional response of these *ND* genes to a large number of stresses, both biotic and abiotic. Taken together, these observations indicate that *NDB3* is the main stress-responsive *ND* gene in rice and barley, further suggesting that it serves a similar functional role as *AtNDB2*.

A more detailed analysis of the response of rice to saline conditions also suggested post-translational regulation of AOX by stress. Transcript abundance of various *AOX* genes, but especially *OsAOX1a* and *OsAOX1d*, gradually increased in shoots but decreased in roots. The results with shoots agree with those of Ohtsu et al. [20] and Feng et al. [21]. In contrast, Feng et al. [51] reported an increase of transcript abundance of *AOX1a* and *AOX1d* when roots were exposed to 200 mM and 300 mM salt stress for 12 h, but their experimental conditions may have caused a “salt shock”, which was avoided in our experiments. Ohtsu et al. [20] also reported an increase in transcript abundance of *OsAOX1a* and *OsAOX1d* under a similar ‘salt shock’ experiment, which then decreased gradually. In our experiments, salt concentration was gradually increased over time with several increments, perhaps resulting in different signals. Microarray data from Genevestigator clearly demonstrate the tissue-specific nature of *OsAOX1* expression in rice in response to salinity stress and the patterns of *OsAOX1a* and *OsAOX1d* expression in various tissues from different cultivars are consistent with our results. In particular, there was a reduction of *OsAOX1a* and *OsAOX1d* transcript abundance in roots from the cultivars Pokkali, IR63731, IR29 and FL478, eight days after salt application (Table S5). Despite this decrease in *AOX* gene transcript over the long term, AOX capacity increased in mitochondria isolated from salt stressed rice root tissue. This may reflect post-translational regulation by reduction of AOX from its inactive oxidised dimer to an active reduced form. Such post-translational regulation of AOX capacity has also been observed during high light stress in the shade plant *Allocasia* [52] and in soybean leaves [53]. Since OsAOX1d has a serine at the Cys_I_ site involved in redox control of AOX activity, which will prevent its conversion to an inactive covalently linked dimer, it is likely that the protein we detected immunologically in roots was another AOX isoform.

The difference in results between the studies of Feng et al. [51], Ohtsu et al. [20] and the present study, suggests that different signals were involved in the roots under the different conditions imposed, one leading to a transcriptional upregulation of *AOX* genes, the other to post-translational regulation of existing protein. This may reflect different roles of AOX in response to a direct oxidative stress (KCN) versus salt stress where it might play a role in metabolite synthesis, but this remains to be proven. However, it does highlight the complexity of regulation of AOX and AP in plants.

Less is known currently about the response of barley AP components to stress. Up-regulation of an *AOX* gene in response to cold has been reported for wild barley [54] and heat stress (*HvAOX1a*) for the commercial cultivar Golden Promise [28]. More recently, Zhao et al. [25] and Wang et al. [26] reported an increase in AOX protein linked to varieties of highland barley showing improved tolerance to low N, UVB and salt. We have undertaken a more detailed analysis of the *AOX* and *ND* gene family to oxidative, abiotic and biotic stresses, building on the previous work and confirming that these transcriptional responses are translated into higher AOX protein levels under drought stress. This suggests that AOX may play a similar role during drought to that shown in tobacco [48]. Whether ND protein increases as well awaits the development of antibodies to detect these proteins.

In conclusion, we have shown that there is a high level of co-expression of *AOX1a* and *AOX1d*, together with *NDB3*, in response to chemical and environmental stresses in rice and barley. This is reminiscent of the co-expression that has been well characterised in Arabidopsis for *AtAOX1a* and *AtNDB2*. Our results pave the way for an assessment of the role the AP path in these important cereal crops plants and the possible use of AP components as molecular markers in breeding programs.

## 4. Materials and Methods

### 4.1. Plant Material

*Oryza sativa* ssp. *japonica*, cv. Nipponbare or *Hordeum vulgare*, cv. Golden Promise were used for transcript level, protein level and enzyme activity analyses as described individually for each experiment.

### 4.2. Gene Identification

Nucleotide and amino acid sequences for all Arabidopsis *AOX*s (*AtAOX1a*, At3g22370; *AtAOX1b*, At3g22360; *AtAOX1c,* At3g27620; *AtAOX1d*, At1g32350; *AtAOX2*, At5g64210) and NDs (*AtNDA1,* At1g07180; *AtNDA2*, At2g29990; *AtNDB1*, At4g28220; *AtNDB2*, At4g05020; *AtNDB3*, At4g21490; *AtNDB4*, At2g20800; *AtNDC1*, At5g08740) were retrieved from the The Arabidopsis Information Resource [55] and used for BLASTp/BLASTn searches against protein/genomic reference sequences in the MSU RGAP Release 7 on Rice Genome Annotation Project database [56] to identify alternative pathway homologs in japonica rice. Putative japonica rice AP genes were then used in BLASTp/BLASTn searches against *O. sativa* ssp. *indica* group sequences on the Gramene database [57] to identify AOX and ND homologs in indica rice. The rice AP sequences (Table S1) were also used for BLASTp/BLASTn searches against reference sequences of genes from NCBI [58] and the International Barley Sequencing Consortium database [59] to identify AP homologs in barley. Protein sizes were determined using the predicted amino acid sequence and the ExPASy tool [60].

### 4.3. Protein Relationship Analyses

A protein relationship tree of NDs from rice, barley and Arabidopsis, was constructed using the neighbour-joining method [61] in the MEGA7 computer program [62]. Protein sequences identified above (Table 1 and Table S1) were aligned in Clustal Omega [63] following standard parameters. Phylogenetic tree construction was carried out using the Neighbour-joining method and Bootstrap of 1000 replications; “number of differences” as the substitution model and “complete deletion” for gaps/missing data treatment.

### 4.4. Subcellular Localization

Computational determination of the subcellular localization of identified AP genes from rice and barley was carried out using publicly available subcellular localization prediction programs; TargetP [64], Predotar [65], ChloroP 1.1 [66] and Plant-mPLOC [67]. Proteins targeting to the peroxisomes were predicted using PredPlantPTS1 [68].

### 4.5. Genevestigator Expression Analyses

Microarray probe-set IDs with high homology were retrieved for all Arabidopsis, rice and barley AP genes, except barley *HvNDB2*, which was not represented on the Affymetrix Barley Genome Array chip. At the time of analysis, the transcriptomic databases for rice and barley contained data for 2656 and 1822 samples, respectively, and featured many different stress experiments. The ‘Perturbation’ dataset was screened using the Condition Search Tool within Genevestigator [69] to assess the stress-responsiveness of each gene in all microarray datasets available on Genevestigator. Fold changes for each AP gene in response to selected biotic and abiotic stress treatments are given in Tables S5 (rice) and S6 (barley). The Similarity Search Tool for Co-expression was also applied to the “Perturbation” dataset to identify genes that may be co-expressed with particular AP genes from Arabidopsis, rice and barley during stress (Table S7).

### 4.6. Screening for Responsive AP Genes under Oxidative Stress

To generate oxidative stress, rice seedlings (*O. sativa* ssp. *japonica*, cv. Nipponbare) were grown on Petri dishes for three weeks, then exposed to the respiratory inhibitor, potassium cyanide (KCN, 5 mM) and barley seedlings (*H. vulgare*, cv. Golden Promise) were grown for two weeks on damp paper towel in petri dishes, then exposed to KCN (5 mM). RNA for gene expression analysis was extracted from shoot and root tissue collected at 6 h and 24 h for both KCN treated and control (no KCN) tissue.

*O. sativa* (cv. Nipponbare) seedlings grown in a hydroponic system, were exposed to salinity stress following transplantation of the seedlings (7–10 days old) to 12 L hydroponic tanks filled with constantly aerated nutrient solution [70], at 26 °C/22 °C (day/night), 12 h photoperiod and 75% relative humidity, 400–500 µE m^−2^·s^−1^. The solution was changed after two weeks and 120 mM salt stress was applied in two increments, 50 mM followed by a further 70 mM NaCl two days later. Supplementary CaCl_2_, 0.75 and 1 mM, was added respectively to keep constant activity of calcium. Salt was applied gradually for several increments, to avoid a salt “shock” and calcium was present to remove any complications from an induced calcium deficiency that can occur in the presence of sodium ions [71]. Root and shoot tissues were harvested from salt-treated and control tanks at pre-determined time points, and either snap-frozen in liquid nitrogen for gene expression analyses or used fresh for mitochondrial isolation and enzyme activity assays.

Drought tolerance assays were carried out using barley seedlings grown in soil. Seedlings (7 days old) of *H. vulgare* (cv. Golden Promise) were transferred from Petri dishes into Osmocote soil in pots. Each pot contained the same dry weight of soil and the same volume of water. The pants were grown in a glasshouse for 7 days at 23 °C/15 °C (day/night), 13 h photoperiod (1500 µmol m^−2^·s^−1^), with equal watering (to weight) before the drought treatment was initiated by completely withholding water for 14 days. Control plants continued to be watered as necessary. Shoot tissues were harvested from drought treated and control plants, immediately snap-frozen in liquid nitrogen and subsequently used for protein extraction and immunoblotting analyses.

### 4.7. Gene Expression Analysis Using qRT-PCR

Samples were snap frozen in liquid nitrogen then stored at −80 °C until use. In experiments with rice, the leaves (two youngest) and root were sampled separately. In experiments with barley the entire shoot and root were harvested separately. Total RNA was extracted using the TRIZOL-like extraction method [72] following recent modifications [71]. Genomic DNA contamination was eliminated by DNase treatment using DNase I (Invitrogen-ThermoFisher, Waltham, MA, USA). Single stranded cDNA synthesis was performed on 1 µg of DNase-treated RNA using iScript cDNA Synthesis Kit (Bio-Rad, Hercules, CA, USA). The qRT-PCR analysis was performed with a CFX96 Real Time PCR Detection System (Bio-Rad). For rice, reactions (10 μL) included SsoFast EvaGreen Supermix Master Mix (Bio-Rad) and 0.6 μM gene-specific primers (Table S8). For barley, reactions (10 µL) included KAPA SYBR-Fast qPCR Universal ReadyMix (Geneworks, Thebarton, Australia) and 0.2 µM gene-specific primers (Table S8).

The gene expression data obtained were normalized using the geometric average expression of three stable reference genes. For rice these were *OsElF1* [70], *OsPplase* [73] and *OsActin* [73]. For barley, these were *HvActin*, *HvUbiquitin* and *HvPdf* (Protein phosphatase) based on their stable expression levels according to data from Genevestigator.

### 4.8. Protein and Enzyme Activity Assays in Isolated Rice Tissue Mitochondria

Mitochondrial isolation was carried out following the method described by Kristensen, et al. [74] with slight modifications. Purification of isolated mitochondria was carried out using a discontinuous Percoll gradient, which consisted of four layers: 6 mL of 60% (*v*/*v*) Percoll, 8 mL of 45% (*v*/*v*) Percoll, 10 mL of 28% (*v*/*v*) Percoll and 10 mL of 5% (*v*/*v*) Percoll.

### 4.9. Oxygen Electrode Assays

The oxygen uptake rate of mitochondria isolated from rice shoots or roots was measured in a standard reaction medium (0.3 M mannitol, 10 mM TES, 10 mM KH_2_PO_4_, 2 mM MgCl_2_, pH 7.2 at 25 °C using a Clark-type Oxygraph Plus oxygen electrode system (Hansatech Instruments Ltd., Pentney, UK). Total oxygen consumption rate of AOX was measured in the presence of the following components; 10 mM succinate, 5 mM pyruvate, 1 mM NADH, 1mM ATP, 1 mM ADP, 5 mM DTT. The cyanide resistant and the residual oxygen consumption rates were determined in the presence of 1 mM KCN and 250 nM OG (Octyl Gallate), respectively.

### 4.10. Immunoblot Analysis

Detection of AP protein levels in salt-treated and control rice shoot and root tissues was carried out on isolated mitochondria resolved on a 10% (*v*/*v*) Tris-glycine SDS-PAGE followed by immunoblotting. For barley tissue samples, protein was extracted from whole shoot or root tissue following the procedure of Umbach, et al. [75]. For both rice mitochondria and barley total protein extracts, samples were prepared either in the presence or absence of 200 mM DTT as indicated. Blots were probed with the monoclonal AOA antibody, with the peptide recognition sequence of RADEAHHRDVNH [76], or anti-porin antibody to show equal loading of samples on gel (supplied by Professor Harvey Millar, University of Western Australia).

The abundance of NDB2 protein was estimated by probing immunoblots with 1:8000 diluted polyclonal antibody OsNDB2, that was prepared by Biomatik (http://www.biomatik.com) with the peptide recognition sequence of CQDNQVLQINDGTGKKR.

### 4.11. Statistical Analyses

Data are generally expressed as mean ± SEM (Standard Error of the Mean). Analysis of variance or *t*-test was performed to determine whether there were significant differences between means of control and treated samples at 95% confidence level using GraphPad Prism (La Jolla, CA, USA).

## Figures and Tables

**Table 1:** Alternative respiratory pathway components identified in barley. *HvAOX1a*, *HvAOX1d1* and *HvAOX1d2* cDNA sequences as well as a *HvAOX1c* EST sequence were identified in Costa et al., 2014. BLAST servers from NCBI (National Centre for Biotechnology Information) and IBSC (International Barley Sequencing Consortium) were used to find barley sequences with high homology with the rice alternative dehydrogenase sequences identified in [3,24]. Chromosome location as well as genomic sequences (contigs) were found using IPBC. * are partial sequences.

Gene	Chromosome Location chr (Chromosome Number) cM (centi-Morgan)	Contig ID (Genomic Sequence/s	Relevant cDNA Accession Number/s	Relevant Protein Accession Number/s (Predicted from cDNA Sequence)	Predicted Protein Size (kDa)
*HvAOX1A*	chr = 2H cM = 105.87 chr2H:695364063-695501552	morex_contig_38523	AK363239	AK363239 (on IBSC) HORVU2Hr1G101980.1	36.36
*HvAOX1C*	chr = 6H cM = 63.67 chr = 6H cM = 63.46 chr6H:472176203-472177532	morex_contig_1576377 morex_contig_1587016	HORVU6Hr1G068150.2 * GH226429.1 * AK251266.1 *	HORVU6Hr1G068150.2 *	36.13
*HvAOX1D1*	chr = 2H cM = 106.86	morex_contig_99492	AK365405.1	BAJ96608.1 HORVU0Hr1G005420.4	36.38
*HvAOX1D2*	chr = 2H cM = 106.86 chr2H:695390581-695392039	morex_contig_242686	AK369112.1	BAK00314.1 HORVU2Hr1G101990.1	37.08
*HvNDA1*	chr = 2H cM = 59.5 chr7H:445843858-445845235	morex_contig_2565566 morex_contig_363823	AK376348.1	BAK07543.1 HORVU7Hr1G076330.1	60.06
*HvNDA2*	chr = 3H cM = 99.58	morex_contig_7977 morex_contig_348032 morex_contig_189545 morex_contig_2081103	AK354319.1	BAJ85538.1 HORVU3Hr1G087850.1 HORVU4Hr1G058410.1 HORVU5Hr1G020920.2 HORVU7Hr1G040660.3 HORVU0Hr1G000930.1 HORVU2Hr1G101830.2 HORVU2Hr1G040130.1	54.21
*HvNDB1*	chr = 7H	morex_contig_1569455	HORVU7Hr1G101500.3	HORVU7Hr1G101500.3	65.08
*HvNDB2*	chr3H: 570908294-570913236	morex_contig_36877	AK367948.1	BAJ99151.1 HORVU3Hr1G076920.2	64.23
*HvNDB3*	chr = 7H cM = 70.61	morex_contig_47987	AK354220.1 AK362739.1 AK371807.1 AK252091.1 AK371114.1	BAJ85439.1 BAJ93943.1 BAK03005.1 BAK02312.1	64.59
*HvNDC1*	chr = 7H cM = 61.76	morex_contig_49824 morex_contig_60206	AK364677.1	AK364677 (on IBSC) HORVU7Hr1G035810.4	59.33

**Table 2:** Expression of rice AP genes under chemical stress. Exposure to potassium cyanide (5 mM) up-regulates *AOX* and *ND* gene expression in rice (*O. sativa* ssp. *japonica*, cv. Nipponbare). Gene expression analyses were carried out using qRT-PCR and transcript levels were determined in both shoot and root tissues exposed to treatments for 6 h and 24 h or grown under control conditions. Data are shown as mean fold change ± SE of three biological replicates relative to the control at each point set at 1.00. * indicates significant transcript level changes relative to the control (*p* ≤ 0.05).

Tissue	*OsAOX1a*	*OsAOX1c*	*OsAOX1d*	*OsNDA1*	*OsNDA2*	*OsNDB1*	*OsNDB2*	*OsNDB3*	*OsNDC1*
6 h shoots	58.2 ± 15.51 *	0.3 ± 0.08	248.6 ± 63.97 *	17.4 ± 3.13 *	0.2 ± 0.04	0.2 ± 0.11	5.2 ± 0.91 *	18.3 ± 0.75 *	0.6 ± 0.07
24 h shoots	1.0 ± 0.29	0.1 ± 0.02	348.6 ± 78.37 *	0.7 ± 0.05	1.1 ± 0.20	0.9 ± 0.66	1.9 ± 0.42	0.7 ± 0.05	0.2 ± 0.11
6 h roots	44.0 ± 4.52 *	1.7 ± 0.25	355.4 ± 46.55 *	56.9 ± 13.04 *	3.0 ± 0.81	1.6 ± 1.13	2.0 ± 0.37	29.7 ± 5.64 *	1.0 ± 0.19
24 h roots	5.1 ± 1.01	0.1 ± 0.02	11.3 ± 1.36	2.0 ± 0.15	6.0 ± 1.33*	0.9 ± 0.23	7.1 ± 1.63 *	1.0 ± 0.38	0.4 ± 0.14

**Table 3:** Expression of barley AP genes under chemical stress. Fourteen-day-old barley seedlings (cv. Golden Promise) were exposed to 5 mM potassium cyanide for 6 and 24 h. Transcript amount was determined using qRT-PCR. Data were normalized with Housekeeping genes, *Actin*, *Ubiquitin* and *Pdf* (Protein phosphatase) and shown as mean fold change ± SE of four biological replicates relative to the control at each point set at 1.00. ND refers to being at the limit of detection for the assay used. After KCN treatment, *HvNDA1* did increase but to a very low level, and the absence of expression in the control tissue made this difficult to express as a fold change. * denotes significantly different (*p* < 0.01) from the control.

Tissue	*HvAOX1a*	*HvAOX1c*	*HvAOX1d1*	*HvAOX1d2*	*HvNDA1*	*HvNDA2*	*HvNDB1*	*HvNDB2*	*HvNDB3*	*HvNDC1*
6 h shoots	16.5 ± 7.2	0.6 ± 0.3	23 ± 5 *	80 ± 14 *	N.D.	26 ± 19	1.4 ± 0.25	2.5 ± 1.0	17 ± 1.8 *	1.2 ± 0.6
24 h shoots	10.1 ± 4.7	0.3 ± 0.1 *	3.5 ± 1.7 *	3.5 ± 1.5	N.D.	1.2 ± 0.1	3.3 ± 1.0	3.2 ± 1.2	2.2 ± 0.5 *	0.1 ± 0.02
6 h roots	27 ± 1.6 *	N.D.	17 ± 3.4 *	14 ± 3 *	N.D.	4.8 ± 1.0	0.8 ± 0.2	1.6 ± 0.3 *	4.5 ± 1.3 *	3.4 ± 1.8
24 h roots	6.7 ± 1.8	N.D.	4.1 ± 1.7	2.2 ± 1.2	N.D.	1.8 ± 0.2 *	0.7 ± 0.1	1.5 ± 0.3	1.7 ± 0.3	0.2 ± 0.14

## Supplementary Materials

Supplementary materials can be found at http://www.mdpi.com/1422-0067/19/3/915/s1.
