# Supplementary material for: Alternative Respiratory Pathway Component Genes (AOX and ND) in Rice and Barley and Their Response to Stress
Source: Int J Mol Sci. 2018 Mar 20;19(3):915. doi: 10.3390/ijms19030915 (PMC5877776; doi:10.3390/ijms19030915)
Supplement: Supplementary file 1 [file ijms-19-00915-s001.pdf]

Table S1. Alternative respiratory pathway genes identified in japonica and indica rice. Four isoforms of type 1 AOX and six NDH were identified in japonica rice as well as in indica rice. Amino acid (aa) sequence alignment studies showed that AOX and NDH from japonica rice were very similar to those from indica rice except NBD3. NDB3 from japonica rice consisted of a shorter sequence compared to NDB3 from indica rice. * NDB3 in japonica is truncated to 357 aa from 580 aa in indica, which may be due to a sequencing error in the database information, Rice Genome Annotation Project (http://rice.plantbiology.msu.edu) and Gramene (http://www.gramene.org).

**Alternative oxidases**						**Alternative dehydrogenases**					
Gene name	Japonica rice		Indica rice		Identity (%)	Gene name	Japonica rice		Indica rice		Identity (%)
	Gene ID	Length (aa)	Gene ID	Length (aa)			Gene ID	Length (aa)	Gene ID	Length (aa)	
OsAOX1a	LOC_Os04g51150	332	BGIOSGA014422	332	100	*OsNDA1*	LOC_Os07g37730	562	BGISOGA025955	561	99.7
OsAOX1c	LOC_Os02g47200	345	BGIOSGA005788	345	100	*OsNDA2*	LOC_Os01g61410	499	BGIOSGA004734	497	99.8
OsAOX1d	LOC_Os04g51160	335	BGIOSGA014421	335	100	*OsNDB1*	LOC_Os06g47000	588	BGIOSGA020655	588	100
OsAOX1e	LOC_Os02g21300	339	BGIOSGA008063	339	99.7	*OsNDB2*	LOC_Os05g26660	575	BGIOSGA018290	575	100
						OsNDB3	LOC_Os08g04630	357*	BGIOSGA027677	580	98.8
						OsNDC1	LOC_Os06g11140	548	BGIOSGA021679	548	99.4

Table S2. Analysis of amino acid sequence identity (%) of ND gene families between **(A)** Arabidopsis and rice, and **(B)** rice and barley

A

	AtNDA1	AtNDA2	AtNDB1	AtNDB2	AtNDB3	AtNDB4	AtNDC1
OsNDA1	71	68					
OsNDA2	78	77					
OsNDB1			66	62	65	61	
OsNDB2			63	60	66	63	
OsNDB3			63	65	68	64	
OsNDC1							64

B

	OsNDA1	OsNDA2	OsNDB1	OsNDB2	OsNDB3	OsNDC1
HvNDA1	74	67				
HvNDA2	70	87				
HvNDB1			86	65	67	
HvNDB2			66	88	75	
HvNDB3			70	73	90	
HvNDC1						87

Table S3. Subcellular localization of identified Type II NADH dehydrogenase (NDH) proteins from rice and barley. Prediction of subcellular targeting of rice alternative respiratory proteins using publicly available subcellular localization prediction programs; TargetP (http://www.cbs.dtu.dk/services/TargetP), Predotar (http://urgi.versailles.inra.fr/Tools), MitoProtII (ihg.gsf.de/ihg/mitoprot.html). Proteins targeting to the peroxisomes were predicted using PredPlantPTS1 (http://ppp.gobics.de).

Protein	Targeting organelle predicted by different programs			
	TargetP	MitoProtII	Predotar	PredPlantPTS1
OsNDA1	Mitochondria	Mitochondria	Mitochondria	Not targeted to Peroxisome
OsNDA2	Mitochondria	Mitochondria	Mitochondria	Peroxisome
OsNDB1	Mitochondria	Mitochondria	Mitochondria	Peroxisome
OsNDB2	Mitochondria	Mitochondria	Mitochondria	Peroxisome
OsNDB3	Mitochondria	Mitochondria	Mitochondria	Not targeted to Peroxisome
OsNDC1	Chloroplast	Mitochondria	Mitochondria	Not targeted to Peroxisome
HvNDA1	Mitochondria	Mitochondria	Mitochondria	Not targeted to Peroxisome
HvNDA2	Mitochondria	Mitochondria	Mitochondria	Peroxisome
HvNDB1	Mitochondria	Mitochondria	Mitochondria	Peroxisome
HvNDB2	Mitochondria	Mitochondria	Mitochondria	Peroxisome
HvNDB3	Mitochondria	Mitochondria	Possibly ER	Not targeted to Peroxisome
HvNDC1	Chloroplast	Mitochondria	Mitochondria	Not targeted to Peroxisome

Table S4. Normalised gene expression values for rice and barley AP genes. For rice, expression data were normalized against the expression of reference gene *OsActin* and shown as mean of three biological replicates. For barley, expression data were normalized to *Actin*, *Ubiquitin* and *Pdf* (protein phosphatase) and shown as mean of 4 biological replicates. These data correspond to the control data used to calculate fold changes presented Tables 2 and 3 and are expressed as fmol/μg RNA.

Gene	Rice				Barley			
	Shoots (Control)		***Roots (Control)***		***Shoots (Control)***		***Roots (Control)***	
	**6 hr**	**24 hr**	**6 hr**	**24 hr**	**6 hr**	**24 hr**	**6 hr**	**24 hr**
AOX1a	14.01 ± 5.22	7.55 ± 3.80	1.27 ± 0.28	1.97 ± 0.40	0.32 ± 0.22	0.10 ± 0.01	0.28 ± 0.05	0.27 ± 0.05
AOX1c	40.95 ± 12.97	19.23 ± 10.24	0.34 ± 0.05	1.09 ± 0.28	0.06 ± 0.00	0.06 ± 0.01	0.0 ± 0.0	0.0 ± 0.0
AOX1d	0.34 ± 0.11	0.36 ± 0.32	1.46 ± 0.08	2.47 ± 0.83				
AOX1d1					0.23 ± 0.04	0.34 ± 0.01	0.66 ± 0.07	0.48 ± 0.12
AOX1d2					0.08 ± 0.02	0.15 ± 0.17	0.21 ± 0.02	0.29 ± 0.04
NDA1	0.851 ± 0.45	1.405 ± 0.80	0.047 ± 0.01	0.102 ± 0.03	0.0 ± 0.0	0.0 ± 0.0	0.0 ± 0.0	0.0 ± 0.0
NDA2	0.006 ± 0.004	0.003 ± 0.001	0.003 ± 0.001	0.001 ± 0.0004	0.68 ± 0.08	1.01 ± 0.18	0.26 ± 0.03	0.26 ± 0.03
NDB1	0.032 ± 0.01	0.028 ± 0.01	0.019 ± 0.01	0.020 ± 0.01	1.01 ± 0.18	1.15 ± 0.05	0.15 ± 0.01	0.27 ± 0.04
NDB2	0.159 ± 0.06	0.109 ± 0.03	0.093 ± 0.02	0.157± 0.11	0.01 ± 0.01	0.01 ± 0.00	0.04 ± 0.00	0.05 ± 0.01
NDB3	0.225 ± 0.08	0.261 ± 0.02	0.052 ± 0.03	0.382 ± 0.06	0.44 ± 0.04	0.55 ± 0.04	0.70 ± 0.08	0.91 ± 0.21
NDC1	0.036 ± 0.014	0.028 ± 0.001	0.004 ± 0.001	0.006 ± 0.001	0.01 ± 0.00	0.07 ± 0.03	0.00 ± 0.00	0.03 ± 0.02

Table S5. Effect of various biotic and abiotic stresses on rice AP gene expression. Presented as fold-changes from a Compendium-Wide Condition Search analysis of the Perturbation dataset within Genevestigator. *Statistically significant fold-changes based on $p < 0.05$ from un-paired *t*-tests.

Experiment	Perturbations	*OSAOX1a*	*OsAOX1c*	*OsAOX1d*	*OsAOX1e*	*OsNDA1*	*OsNDA2*	*OsNDB1*	*OsNDB2*	*OsNDB3*	*OsNDC1*
OS-00103	cold study 5 (LTH; 4°C for 48h) / untreated leaf samples (LTH)	38.05*	3.59*	79.40*	5.63*	48.40*	2.02*	6.97*	17.83*	35.53*	1.00*
OS-00011	*M. grisea* study 1 (4dpi) / mock treated leaf samples (4dpi)	33.89*	4.87*	100.15*	1.47*	76.43*	0.84	0.91	20.48*	68.26*	1.02*
OS-00051	anoxia study 2 (N2 for 24h; air for 6h) / anoxia study 2 (N2 for 24h)	23.44*	1.07	10.20*	1.98*	6.80*	4.17*	1.67*	12.47*	12.05*	1.00*
OS-00051	anoxia study 2 (N2 for 24h; air for 3h) / anoxia study 2 (N2 for 24h)	22.96*	1.03	1.21	1.38*	5.55*	3.12*	1.64*	11.25*	8.54*	1.03*
OS-00103	cold study 5 (LTH; 4°C for 24h) / untreated leaf samples (LTH)	15.58*	2.48	36.28*	4.07*	23.10*	1.93	2.86*	16.14*	21.13*	1.00*
OS-00041	drought study 2 (leaf; panicle elongation) / untreated leaf samples (panicle elongation)	12.58*	12.05*	29.28*	1.11	3.64*	2.31	3.24*	4.72*	13.10*	1.01*
OS-00102	cold study 4 (C418; 48h) / untreated shoot samples (C418)	10.51*	1.61*	28.54*	2.13*	25.92*	1.62*	1.84*	2.36*	6.78*	1.06
OS-00070	cold study 2 (Huahui 1) / untreated leaf samples (Huahui 1)	10.32*	1.43*	7.77*	1.24	18.06*	1.25*	1.17*	3.82*	4.11*	1.01*
OS-00096	phosphorus study 3 (35S::PSTOL1 (20); no P fertilizer) / phosphorus study 3 (35S::PSTOL1 (20); 60kg/ha P2O5)	9.30*	12.35*	16.59*	1.24*	50.63*	2.56*	3.12*	5.27*	10.26*	1.01*
OS-00070	cold study 2 (MH63) / untreated leaf samples (MH63)	8.85*	1.34*	6.22*	1.23	17.47*	1.19	1.15	3.22*	3.26*	1.06
OS-00003	arsenate study 2 (Azucena) / untreated root samples (Azucena)	8.81*	1.81	34.34*	2.49*	8.74*	0.87	0.98	9.49*	2.99*	1.08
OS-00096	phosphorus study 3 (IR64-null; no P fertilizer) / phosphorus study 3 (IR64-null; 60kg/ha P2O5)	8.62*	8.72*	14.32*	1.06	34.73*	2.36*	2.50	3.70	9.33*	1.03*
OS-00103	cold study 5 (IR29; 4°C for 48h; 29°C for 24h) / untreated leaf samples (IR29)	8.32*	2.75*	14.33*	3.70*	10.87*	1.77*	1.65	15.63*	9.72*	1.10
OS-00070	heat study 2 (Huahui 1) / untreated leaf samples (Huahui 1)	7.72*	1.30*	5.89*	1.18	12.13*	1.11*	1.11	1.28	3.08*	1.02*
OS-00041	drought study 2 (root; panicle elongation) / untreated root samples (panicle elongation)	7.68*	5.72*	18.32*	1.06	2.60*	1.91*	2.30	2.88*	5.89*	1.00*
OS-00070	heat study 2 (MH63) / untreated leaf samples (MH63)	6.84*	1.22*	5.77*	1.15	11.08*	1.11	1.09	1.28*	2.23*	1.03*
OS-00103	cold study 5 (LTH; 4°C for 8h) / untreated leaf samples (LTH)	6.30*	1.82*	8.10*	2.28*	6.94*	1.78	1.08	6.36*	4.60*	1.85
OS-00102	cold study 4 (K354; 48h) / untreated shoot samples (K354)	6.27*	1.62*	7.13*	1.23	10.89*	1.58*	1.16	2.28*	3.39*	1.08
OS-00008	salt study 2 (3h) / untreated seedling samples (3h)	5.78*	2.40*	5.49*	3.86*	26.05*	1.76	0.87	2.40*	8.55*	1.89
OS-00037	*A. tumefaciens* study 1 (6h) / untreated calli samples	5.06*	5.24*	14.34*	4.32	32.99*	2.03	1.77	11.95*	16.52*	1.14
OS-00040	Cr(VI) study 1 (24h) / untreated root samples	5.05*	1.13	13.11*	0.86	20.78*	1.68	0.87	4.76*	2.04*	1.75
OS-00103	cold study 5 (LTH; 4°C for 2h) / untreated leaf samples (LTH)	4.93*	1.55	5.05*	1.60*	7.22*	1.32	0.85	3.60*	3.90*	1.95
OS-00074	A. tumefaciens study 2 (Nipponbare; 1h) / untreated calli samples (Nipponbare)	4.75*	2.21*	20.72*	2.06	18.35*	1.86*	1.67*	10.01*	8.20*	1.02*
OS-00073	*N. lugens* study 1 (Taichung Native-1; 8hpi) / untreated stem samples (Taichung Native-1)	4.37*	1.30	2.79	1.14	11.95	1.22	1.42	0.98	1.49	1.02*

OS-00061	*X. oryzae* pv. oryzae study 4 (Nipponbare; T7174) / mock treated leaf samples (Nipponbare)	4.29*	3.18*	4.19*	4.82*	10.49*	1.62*	1.91*	4.76*	27.63*	1.03*
OS-00008	dehydration study 1 (3h) / untreated seedling samples (3h)	4.01*	1.73*	2.88*	1.72*	14.05*	1.44*	0.78*	1.64	4.50*	1.48
OS-00119	salicylic acid study 1 (3h) / mock treated seedling samples (3h)	3.35	4.83*	4.27	6.51*	6.04	2.41*	1.41*	2.92	3.43*	1.08
OS-00119	ABA study 2 (3h) / mock treated seedling samples (3h)	3.33*	1.24	3.28*	5.07*	2.39	1.37	0.86	2.85	2.81	1.20
OS-00072	*M. grisea* study 2 (Taipei 309) / mock treated leaf samples (Taipei 309)	3.28*	5.78*	1.21	1.03	0.82	4.61*	1.00	1.48	2.08*	1.01*
OS-00123	dehydration study 2 (Dongjin) / untreated root samples (Dongjin)	2.81	2.67	5.31	2.61	11.03*	1.60	2.15	2.56	4.54	1.45
OS-00024	heat study 1 (3h) / untreated seedling samples (3h)	2.59*	2.64*	4.53*	0.64*	2.19*	1.58*	1.07	3.50*	3.18*	1.02*
OS-00041	drought study 2 (root; tillering) / untreated root samples (tillering)	2.32*	3.00*	6.42*	0.84	1.32	1.48*	1.40*	1.25	4.02*	1.54
OS-00015	IAA study 1 (1+3h) / mock treated seedling samples (1+3h)	2.15	1.16*	3.08*	5.24*	3.73*	1.09	1.06	1.15	1.22	1.01*
OS-00001	salt study 1 (IR29) / untreated growing point samples (IR29)	1.04	1.08	1.20	1.03	1.52*	1.06	0.98	1.13*	1.42	1.09
OS-00068	drought study 4 (Zhenshan 97; leaf RWC 65-69%) / untreated flag leaf samples (Zhenshan 97; leaf RWC 94-95%)	0.97	3.64*	1.36*	1.23	1.12	1.28	1.24*	1.33*	1.85*	1.00*
OS-00025	salt study 5 (Pokkali) / untreated root samples (Pokkali)	0.32*	0.91	0.28	0.58*	0.27*	0.69	1.07	0.92	0.50*	1.23
OS-00023	salt study 3 (FL478) / untreated root samples (FL478)	0.26*	1.01	0.19*	0.68*	0.46*	0.72	0.74	1.06	0.79	1.06

Table S6. Effect of various biotic and abiotic stresses on barley AP gene expression. Presented as fold-changes from a Compendium-Wide Condition Search analysis of the Perturbation dataset within Genevestigator. *Statistically significant fold-changes based on $p < 0.05$ from un-paired *t*-tests.

Experiment	Perturbations	HvAOX1a	HvAOX1c	HvAOX1d1	HvAOX1d	HvNDA1	HvNDA2	HvNDB1	HvNDB3	HvNDC1
HV-00057	malting study 2 (Morex; germinated) / malting study 2 (Morex; steeped)	28.56*	0.42*	19.78*	56.57*	7.97*	0.45*	0.87*	50.11*	0.50*
HV-00020	B. graminis study 1 (rom1; 0+8+16hpi) / non-infected rom1 leaf samples (0+8+16h)	27.02*	1.36	38.35*	23.23*	8.33*	1.18	1.06	6.68*	1.18
HV-00025	B. graminis study 3 (m11542(bcd1); 5874; 16hpi) / B. graminis study 3 (m11542(bcd1); K1; 16hpi)	8.21*	0.75*	6.13*	5.31*	1.07	0.88	1.07	5.34*	0.90
HV-00006	B. graminis study 2 (m9472; 20+24hpi) / non-infected m9472 leaf samples (20+24h)	7.07*	1.46	18.00*	11.50*	1.52*	1.89*	1.20	11.00*	1.34
HV-00025	cold study 1 (chilling; freeze-thaw) / untreated shoot samples	7.01*	0.67	6.08*	5.11*	0.87	0.88*	0.88	4.43*	0.52*
HV-00026	B. graminis study 1 (mla12-M66; 0+8+16hpi) / non-infected mla12-M66 leaf samples (0+8+16h)	4.67	1.05	28.56*	19.12*	1.05	1.05	0.97	12.62*	0.86
HV-00035	B. graminis study 3 (C.I. 16155(Mla13); K1; 24hpi) / B. graminis study 3 (C.I. 16155(Mla13); 5874; 24hpi)	4.66*	0.65	1.26	1.15	0.97	0.94	1.01	0.97	2.59*
HV-00006	cold study 2 (xan-b12) / untreated xan-b12 leaf samples	4.63*	1.21	8.66	5.83*	1.26*	1.60*	1.13	6.85*	1.16
HV-00025	drought study 1 (OWB-R) / untreated seedling samples (OWB-R)	4.60*	0.23*	4.92*	2.76*	0.78*	0.46	0.81*	3.66*	0.42*
HV-00006	deoxynivalenol study 1 (48h) / mock treated floret samples (48h)	4.34*	1.19	7.97*	5.53*	1.26*	1.56*	1.13	6.77*	1.14
HV-00006	P. graminis study 1 (Golden Promise; QCC; 18+24hpi) / non-infected leaf samples (Golden Promise; QCC; 0hpi)	4.05*	1.17	6.28	4.61*	1.23	1.51*	1.09	6.44*	1.13
HV-00053	cold study 3 (4°C for 49d; 20°C for 1d) / untreated seedling samples	3.79*	0.94	10.84*	2.94*	1.04	1.14*	0.97	12.81*	1.00
HV-00025	B. graminis study 2 (C.I. 16137(Mla1); 20+24hpi) / non-infected C.I. 16137(Mla1) leaf samples (20+24h)	3.67*	0.23*	4.56*	2.56	0.75*	0.45*	0.80*	3.45*	0.39*
HV-00005	F. graminearum study 2 (Z3639; 96hpi) / mock inoculated spike samples (96hpi)	3.41*	1.15	6.98*	7.82*	1.04	1.04	1.02	7.13*	1.10
HV-00006	F. graminearum study 1 (72+96+144hpi) / mock inoculated spike samples (72+96+144hpi)	3.33*	1.14	6.08	4.37*	1.16	1.48*	1.08	6.32*	1.11
HV-00035	deoxynivalenol study 1 (1h) / mock treated floret samples (1h)	3.17*	0.54*	0.99	0.80	0.93	0.67	0.91	0.77	1.99
HV-00005	deoxynivalenol study 1 (24h) / mock treated floret samples (24h)	3.08*	1.13	6.76*	7.27*	1.02	1.01	1.00	6.25*	1.03
HV-00006	F. graminearum study 2 (Z3639; 48hpi) / mock inoculated spike samples (48hpi)	2.84*	1.09	5.59	3.90	1.13	1.38*	1.06	5.78*	1.05
HV-00020	drought study 3 (Martin; 3d at 10% AWC) / drought study 3 (Martin; drop to 10% AWC)	2.50*	0.88	3.90*	2.43*	0.96	0.77	0.94	1.40	0.92
HV-00046	ABA study 1 (1+2+4h) / ABA study 1 (0h)	2.40*	1.26*	1.16*	1.61*	1.23*	1.02	1.13	2.04	1.14
HV-00055	malting study 2 (Cree; germinated) / malting study 2 (Cree; steeped)	2.38*	1.08	1.91*	2.97	1.05	1.09	1.02	1.82	0.98
HV-00055	malting study 2 (M46; germinated) / malting study 2 (M46; steeped)	2.36*	1.07	1.88	2.38	1.04	1.08	1.02	1.76	0.95

HV-00047	P. indica study 1 (19d 12h) / mock inoculated Golden Promise leaf samples (19d 12h)	2.34*	1.14	2.52	1.81	1.19	1.19	1.10	1.31	1.19
HV-00006	cold study 2 (xan-s46) / untreated xan-s46 leaf samples	2.27	1.07	4.81	3.73	1.09	1.35*	1.04	5.31*	1.01
HV-00006	drought study 4 (lemma) / untreated lemma samples	2.02	1.02	3.46	3.12	1.06	1.18	1.01	4.58*	0.96
HV-00047	ABA study 2 / mock treated aleurone samples (Himalaya)	2.01	1.11	2.16	1.61	1.11	1.12	1.07	1.28	1.13
HV-00047	drought study 3 (Moroc9-75; 5d at 10% AWC) / drought study 3 (Moroc9-75; drop to 10% AWC)	1.98	1.11	2.12*	1.58	1.11	1.11	1.07	1.28	1.13
HV-00047	drought study 2 (SWC 19%) / untreated crown samples (SWC 91%)	1.94*	1.10	1.86	1.56	1.10	1.10	1.06	1.23	1.13
HV-00047	heat study 1 (0.5h) / untreated seed samples (0.5h)	1.74*	1.08	1.60	1.46	1.08	1.07	1.04	1.17	1.08
HV-00047	drought study 3 (Moroc9-75; 1d at 10% AWC) / drought study 3 (Moroc9-75; drop to 10% AWC)	1.72	1.07	1.57	1.45	1.08	1.07	1.04	1.16	1.07
HV-00006	cold study 2 (alb-e16) / untreated alb-e16 leaf samples	1.70	0.91	2.38	2.24*	1.03	1.06	0.97	2.63	0.89
HV-00006	boric acid study 1 (10mM) / boric acid study 1 (10µM)	1.69	0.91	2.35	2.21*	1.03	1.06	0.97	2.58	0.88
HV-00047	cold study 1 (chilling; freeze-thaw; chilling; 20°C) / untreated shoot samples	1.68*	1.07	1.52	1.43	1.07	1.06	1.03	1.15	1.06
HV-00047	heat study 1 (6h) / untreated seed samples (6h)	1.66	1.07	1.51	1.43	1.07	1.06	1.03	1.15	1.06
HV-00005	cold study 2 (alb-f17) / untreated alb-f17 leaf samples	1.59*	0.93	2.88*	3.11*	0.94	0.88	0.93	3.47*	0.88*
HV-00047	drought study 3 (Martin; 1d at 10% AWC) / drought study 3 (Martin; drop to 10% AWC)	1.57	1.05	1.42	1.38	1.05	1.05	1.03	1.14	1.05

Table S7. Identification of potential gene co-expression partners for AP genes in Arabidopsis, rice and barley. *AtAOX1a*, *AtNDB2*, and all available rice and barley AP genes were used as queries within the Similarity Search Tool for Co-expression using Genevestigator. The Perturbation datasets were used for each species, to identify genes with positively-correlated gene expression patterns for each AP gene during stress. The search was limited to the 400 top correlated genes, ranked from highest-to-lowest based on the Pearsons correlation coefficient (i,e, a ranking of 1 indicates that this gene demonstrated the highest correlation with the gene of interest, relative to all other genes in the database). Rankings are presented for other AP genes only.

**Target AP gene**	**Co-expressed AP gene**	**Rank**
	**Rice**	
OsAOX1a	*OsAOX1d*	1
	OsNDA1	106
	OsNDB2	Not found
	OsNDB3	Not found
OsAOX1d	*OsAOX1a*	5
	OsNDA1	124
	OsNDB3	125
	OsNDB2	Not found
OsNDA1	*OsAOX1d*	21
	OSAOX1a	23
	OsNDB3	77
OsNDB3	No AP gene found	
OsNDB2	No AP gene found	
OsNDB1	No AP gene found	
	**Barley**	
HvAOX1a	*HvAOX1d1*	8
	HvAOX1d2	14
	HvNDB3	154
HvAOX1d1	*HvAOX1d2*	1
	HvNDB3	11
	HvAOX1a	210
HvAOX1d2	*HvAOX1d1*	1
	HvAOX1a	53
	HvNDB3	58
HvNDB3	*HvAOX1d1*	8
	HvAOX1d	14
	HvAOX1a	207
HvNDB2	No AP gene found	
HvNDA1 or *2*	No AP gene found	

**Arabidopsis**		
AtAOX1a	*AtNDB2*	6
	AtAOX1d	83
AtNDB2	*AtAOX1a*	9
	AtNDA2	10
	AtAOX1d	156

Table S8. Primers used for qRT-PCR assays of rice and barley AP genes.

Gene name	Forward primer sequence	Tm (°C)	Reverse primer sequence	Tm (°C)	Expected product size (bp)
OsAOX1a	CGTCAATCACTTCGCATCGG	53.8	GCAAATCCTCGGCAGTAGAC	53.8	105
OsAOX1c	AAGATCGAGAACGTCCCCGCG	58.3	TCCATCCCCTGAAAATGAACAT	51.1	158
OsAOX1d	AGGGGATGAAGCTGAAGGAT	51.8	TACGCATGTAGTAGCAGTGAT	50.5	109
OsNDA1	GGTGGACCTGAGGGAGAACAAG	58.6	CAGTTGACCGCCACGTAGAACC	58.6	132
OsNDA2	GATGTTAAACAGCGTTATTCACATGT	53.2	ACCTCTTCTCCATTGTCCAGGAT	55.3	197
OsNDB1	TGTGGATTCCCAAGTTAAAAGT	49.2	AATTGTGTCGTCCTGATCCTGT	53.0	163
OsNDB2	AGGATCAGAGGCACAGGGCGTC	60.4	TGGCATAGACAGAATACCATAG	51.1	165
OsNDB3	ATTTGCTGAGGATAAGTTTGGA	49.2	CAATTTGTTTCATGAACTCTGA	47.4	191
OsNDC1	CTACTATTGGAACTTCAACCTGCT	54.0	CTACCTGGCCCCGACCATTTAA	56.7	157
HvAOX1a	CGTCAACCACTTCGCATCGG	65.13	GCCCTCATTTCCTCGGAAGC	64.08	168
HvAOX1c	CGTCCCGTCACCCTCTCAG	61.70	CCGAAGATGACGAGCCTGC	60.88	107
HvAOX1d1	CACTACGCATCCGACATCCA	60.52	GCAACAATCCATCCAAATTAACG	62.14	176
HvAOX1d	CCACTACGCATCCGACATCC	61.58	AGCGGTGGACATAGCAGGC	61.46	145
HvNDA1	GAACCACATGGTGTTCACGC	60.04	GAAGTATGATCCGGGGCGAG	60.04	124
HvNDA2	GGCTATCAACTGGCTGACCA	58.98	GAGAGAGGAGCAGGAGGGAA	58.85	162
HvNDB2	CGTCCACTGTCGCTCTGC	57.52	GGCATCCTCCACTTCCTTCAG	60.99	160
HvNDB2	TTTGGAAGGGACTCCAGCAG	60.66	TCGTTCGGCATATCGTAACA	58.2	193
HvNDB3	GCAAAATCCAGCTACTGGCG	59.90	TTCACGCACCCTTAGCCATT	59.96	157
HvNDC1	ATGGGCCGCCAATTAACTCA	62.7	AACTATCCCGACAGCGACAC	57.36	138

Figure S1. Identification of additional intron in the sequence of *OsNDB3*, japonica rice, with following predicted OsNDB3 polypeptide of 580 aa, correcting the annotated size of the polypeptide for 357 aa. **(A)** Clustal alignment of two annotated japonica rice accessions (LOC_Os08g04630 and Os08t0141400) and indica rice accession BGIOSGA027677. The position of the missing intron is indicated by arrows; **(B)** BLASTN results of the genetic region with the additional intron against rice ESTs from NCBI database with the list of annoted EST accessions;

A

```
LOC_Os08g04630      TTATGGAATGGCTGTTTGGTCCACTGGTATTGGTACCCGGCCCTTCATTTCAGAGTTCAT
Os08t0141400        TTATGGAATGGCTGTTTGGTCCACTGGTATTGGTACCCGGCCCTTCATTTCAGAGTTCAT
BGIOSGA027677       TTATGGAATGGCTGTTTGGTCCACTGGTATTGGTACCCGGCCCTTCATTTCAGAGTTCAT
                    ************************************************************

LOC_Os08g04630      GAAACAAATTGGGCAGGTACCTAGCAAGAAACTCAGCTCGACTCTTTGTAGCTAGCTGAG
Os08t0141400        GAAACAAATTGGGCAGGTACCTAGCAAGAAACTCAGCTCGACTCTTTGTAGCTAGCTGAG
BGIOSGA027677       GAAACAAATTGGGCA---------------------------------------------
                    ***************

LOC_Os08g04630      TTATCCATCTAATTCTCCGTCTACATTTGCAGGGCAAACGGCGTGTACTAGCCACTGATG
Os08t0141400        TTATCCATCTAATTCTCCGTCTACATTTGCAGGGCAAACGGCGTGTACTAGCCACTGATG
BGIOSGA027677       -------------------------------GGGCAAACGGCGTGTGCTAGCCACTGATG
                                                   *************** *************

LOC_Os08g04630      AATGGCTAAGGGTCCGTGAATGTGATGGTGTTTATGCTGTAGGAGATTGTGCTACGATAA
Os08t0141400        AATGGCTAAGGGTCCGTGAATGTGATGGTGTTTATGCTGTAGGAGATTGTGCTACGATAA
BGIOSGA027677       AATGGCTAAGGGTCCGTGAATGTGATGGTGTTTATGCTGTAGGAGATTGTGCTACGATAA
                    ************************************************************
```

B

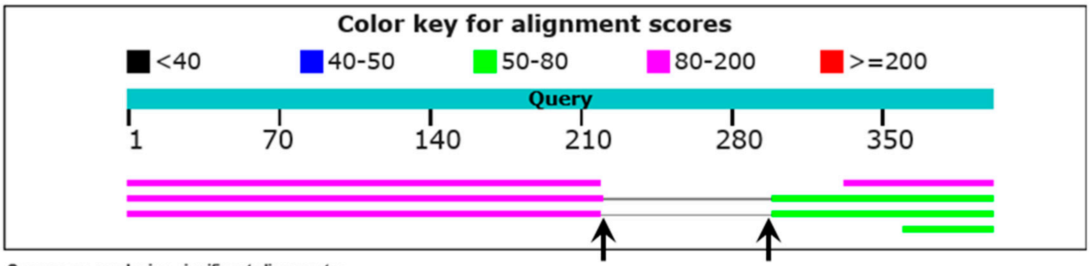

Sequences producing significant alignments:

Select: All None Selected:0

Alignments Download GenBank Graphics Distance tree of results

	Description	Max score	Total score	Query cover	E value	Ident	Accession
☐	SSH002247 Osmotic stress SSH library Oryza sativa Japonica Group cDNA, mRNA sequence	114	114	17%	5e-24	97%	EX450660.1
☐	OSJNEb02K09.f OSJNEb Oryza sativa Japonica Group cDNA clone OSJNEb02K09 5', mRNA sequence	111	170	80%	7e-23	71%	CB642453.1
☐	78719rsicen_23722.y1 Oryza sativa cv. LYP9 booting whole plant cDNA library Oryza sativa Indica Group c	107	166	80%	8e-22	71%	CK076765.1
☐	OSJNEe09G17.f OSJNEe Oryza sativa Japonica Group cDNA clone OSJNEe09G17 5', mRNA sequence	104	104	54%	1e-20	71%	CB674392.1
☐	SSH001241 Osmotic stress SSH library Oryza sativa Japonica Group cDNA, mRNA sequence	71.6	71.6	10%	6e-11	98%	EX450367.1
